# Synaptic and genomic responses to JNK and AP-1 signaling in *Drosophila *neurons

**DOI:** 10.1186/1471-2202-6-39

**Published:** 2005-06-02

**Authors:** Paul D Etter, Radhakrishnan Narayanan, Zaneta Navratilova, Chirag Patel, Dirk Bohmann, Heinrich Jasper, Mani Ramaswami

**Affiliations:** 1 Department of Molecular & Cellular Biology, University of Arizona, Tucson, USA; 2 Department of Brain and Cognitive Sciences, MIT, Cambridge, USA; 3 Department of Biomedical Genetics, University of Rochester, Rochester, USA; 4 ARL Division of Neurobiology, University of Arizona, Tucson, USA

## Abstract

**Background:**

The transcription factor AP-1 positively controls synaptic plasticity at the *Drosophila *neuromuscular junction. Although in motor neurons, JNK has been shown to activate AP-1, a positive regulator of growth and strength at the larval NMJ, the consequences of JNK activation are poorly studied. In addition, the downstream transcriptional targets of JNK and AP-1 signaling in the *Drosophila *nervous system have yet to be identified. Here, we further investigated the role of JNK signaling at this model synapse employing an activated form of JNK-kinase; and using Serial Analysis of Gene Expression and oligonucleotide microarrays, searched for candidate early targets of JNK or AP-1 dependent transcription in neurons.

**Results:**

Temporally-controlled JNK induction in postembryonic motor neurons triggers synaptic growth at the NMJ indicating a role in developmental plasticity rather than synaptogenesis. An unexpected observation that JNK activation also causes a reduction in transmitter release is inconsistent with JNK functioning solely through AP-1 and suggests an additional, yet-unidentified pathway for JNK signaling in motor neurons. SAGE profiling of mRNA expression helps define the neural transcriptome in *Drosophila*. Though many putative AP-1 and JNK target genes arose from the genomic screens, few were confirmed in subsequent validation experiments. One potentially important neuronal AP-1 target discovered, *CG6044*, was previously implicated in olfactory associative memory. In addition, 5 mRNAs regulated by RU486, a steroid used to trigger conditional gene expression were identified.

**Conclusion:**

This study demonstrates a novel role for JNK signaling at the larval neuromuscular junction and provides a quantitative profile of gene transcription in *Drosophila *neurons. While identifying potential JNK/AP-1 targets it reveals the limitations of genome-wide analyses using complex tissues like the whole brain.

## Background

Conserved neuronal signaling pathways regulate synaptic plasticity, the ability of neurons to modify synaptic connections. "Long-term" forms of neuronal plasticity require new gene expression that results in persistent synaptic change (altered synaptic strength and morphology). Thus, long-term forms of plasticity may be perturbed, in a variety of model systems, by protein synthesis inhibitors, or manipulation of either specific signaling kinases or critical downstream transcription factors [1-4].

A major requirement in long-term plasticity for the ERK/MAPK (extracellular signal-regulated kinase subfamily of mitogen-activated protein kinases) signaling cascade acting through CREB (the cAMP response element binding protein, a basic leucine zipper – bZIP – transcription factor) has been supported in diverse experimental paradigms [1,4,5]. Activation of CREB has been reported to enhance long-term memory in *Drosophila *and rodents, and long-term facilitation in the sea slug *Aplysia *[6-11]. Normal ERK signaling is required for hippocampal LTP formation, for BDNF-induced forms of structural plasticity, as well as for several forms of learning and long-term memory [12-15]. For example, ERK activation is necessary for the formation of conditioned taste aversion and spatial learning in rodents, and blockade of ERK signaling affects long-term, but not short-term, fear conditioning [16-18]. In addition, ERK regulates synapse plasticity in flies and LTF and memory in *Aplysia *[19-21].

Many "immediate-early genes" (IEGs), including members of the AP-1 family of transcription factors (heterodimeric transcription factor complexes consisting of the bZIP proteins Fos and Jun), are induced in response to diverse stimuli in the brain, such as electrical stimulation, stress, psychotropic drugs, novel experience and spatial learning [22,23]. Induction of AP-1 mRNA in neurons requires CREB activation [24]. Although roles have been established for AP-1 components Δ*FosB *and *c-fos *in synaptic and behavioral plasticity [25-28], the specific mechanisms and signal transduction pathways that initiate and sustain AP-1 dependent neuronal processes have yet to be elaborated [29]. For instance, the requirement for kinase-mediated modification of immediate-early transcription factors remains poorly studied in the context of neural plasticity, and early-response genes downstream of these critical IEGs, have not yet been identified.

While the majority of plasticity studies have focused on CREB and the ERK signaling cascade [5,30,31], recent studies, especially of other MAP-kinase family members [32], lead to a broader view of the molecules involved in neuronal plasticity and memory formation. The JNK/MAPK (Jun N-Terminal kinase) signaling cascade and AP-1 proteins have recently been shown to play critical roles in long-term plasticity and memory formation in mammals [22,26-28,33]. Similarly, p38/MAPK mediates memory formation in the rat hippocampus [34] and both short- and long-term synaptic depression in *Aplysia *[32].

As suggested by its importance in the control of processes underlying cocaine addiction [29], new data indicate that AP-1 may widely influence transcriptional events that underlie long-term synaptic plasticity. AP-1, under regulation by JNK, positively regulates both synaptic growth and synaptic strength at the *Drosophila *neuromuscular junction (NMJ) [35]. At this synapse AP-1 shows a wider range of influence than CREB whose effects at the same synapse are limited to controlling synaptic strength [36]. Thus, while neural induction of Fos and Jun together is sufficient to cause increases in synaptic size and efficacy at the NMJ, CREB activation, apparently dispensable for synaptic growth, is only essential for AP-1 induced changes in synaptic strength [35-37]. In other neural contexts, the exact roles and mechanisms of AP-1 and JNK signaling in long-lasting forms of plasticity are largely unknown [26-28,33,35,38]. While many genes regulated during CREB and ΔFosB (a splice variant of the *FosB *gene) mediated cocaine reward [39] have been recently identified, this study identified exclusively late-response genes whose expression levels were altered following 5-days to 8-weeks of either ΔFosB and CREB overexpression in the nucleus accumbens, or cocaine injection.

Here, we address two outstanding questions regarding JNK and AP-1 function in synaptic change. First, using temporally controlled induction of a JNK-activating kinase in the fly nervous system, we address synaptic consequences of JNK activation at the *Drosophila *neuromuscular junction. Second, using DNA microarray or SAGE (serial analysis of gene expression) to profile neuronal transcripts in control and experimental animals, we identify groups of neuronal genes potentially regulated by either: a) JNK, or b) AP-1 in the fly CNS within 6 hours of pathway activation. Some of these changes were confirmed by quantitative real-time RT-PCR (Q-PCR), including *CG6044*, that was previously identified in a screen as a potential gene required for normal memory formation in *Drosophila *[40]. We found five genes are responsive to the progesterone-related steroid RU486 commonly used for temporal control of GAL4-restricted transgene expression in *Drosophila *[35,41]. In addition, the *mini-white *gene, a common marker used in most *Drosophila *transgenes, is induced strongly by AP-1 and JNK signaling. These observations and their wider implications are discussed below.

## Results

### Neuronal JNK activation triggers synaptic growth

To assess the effect of neuronal JNK activation on synaptic change we expressed an activated JNK-kinase – *hemipterous *(*hep*^act^) [42] – in the nervous system and analyzed associated phenotypic consequences. Chronic overexpression of *hep*^act ^with neuronal GAL4 drivers (C155, C380, D42 and OK6) caused lethality ranging from late embryonic to early 2^nd ^instar larval stages.

**Figure 1:**
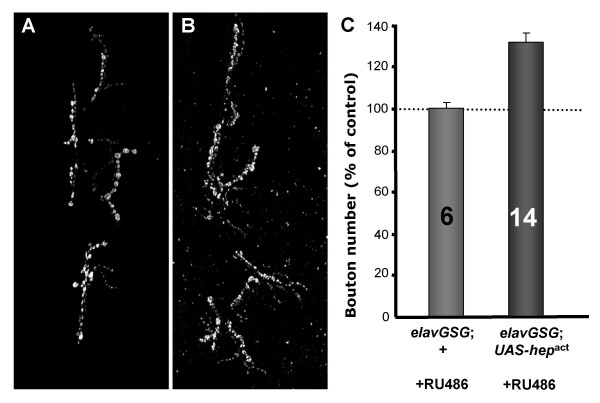
**Postembryonic expression of *hep*^act ^in larval neurons increases synaptic growth. **Confocal projections of synaptic arbors show that synapse size is increased after *hep*^act ^induction in postembryonic neurons (B) compared to control (A). C) A histogram representation of bouton number shows that *hep*^act ^overexpression leads to a 31% increase in synapse size (*P *< 0.001), while exposing wild-type larvae to the inducible ligand does not cause a significant change in synapse size.

In order to overcome this deleterious effect, we drove expression using the inducible GeneSwitch GAL4 (GS-GAL4) system to express *hep*^act ^acutely in postembryonic neurons [35,41]. Age-selected larvae were exposed to the inducing ligand RU486 between late 1^st ^instar and early 2^nd ^instar larval stages and allowed to develop to climbing 3^rd ^instar larval stage. Neural overexpression of *hep*^act ^resulted in a 30% increase in bouton number at the larval NMJ compared to the non-RU486 fed sibling controls (Figure 1). Changes in synapse size may not be attributed to the effect of RU486 since wild-type animals fed the steroid did not show a significant change in bouton number (Figure 1C). Thus, postembryonic activation of JNK signaling in the CNS leads to synaptic growth at the larval motor synapse.

### JNK activation disrupts transmitter release and alters presynaptic composition

**Figure 2:**
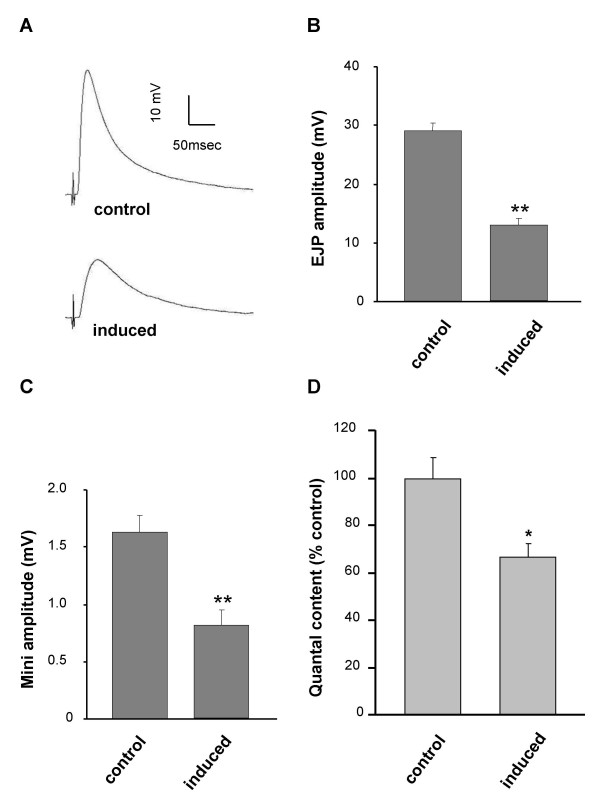
***hep*^act ^expression leads to decreased transmitter release. **A) EJP traces from larvae in which *hep*^act ^expression is induced (lower trace) or control (upper trace). Expression of *hep*^act ^in postembryonic neurons leads to decreases in EJP and miniature (m)EJP amplitude by nearly 50% (*P *< 0.01 for both) compared to control (B, C). D) Quantal content of presynaptic transmitter release is reduced by 35% after *hep*^act ^induction in postembryonic neurons (*P *< 0.04).

To evaluate the effect of JNK activation on transmitter release and postsynaptic response, we measured both spontaneous and evoked junctional potentials with and without JNK activation in postembryonic CNS. Increased *hep*^act ^resulted in an unexpected 60% decrease in the amplitude of excitatory junctional potentials (EJP) (Figure 2A, B). Increased neuronal JNK signaling also decreased the amplitude of spontaneous responses by ~50% (Figure 2C). The quantal content of presynaptic transmitter release shows a 30% decrease when *hep*^act ^is overexpressed in postembryonic neurons (Figure 2D). Therefore, while sustained postembryonic JNK activation in the CNS triggers synaptic growth, the strength of the synapse is reduced. A potential cellular mechanism that underlies this reduction in quantal content was suggested by immunohistochemical analyses of NMJs in JNK-activated animals.

**Figure 3:**
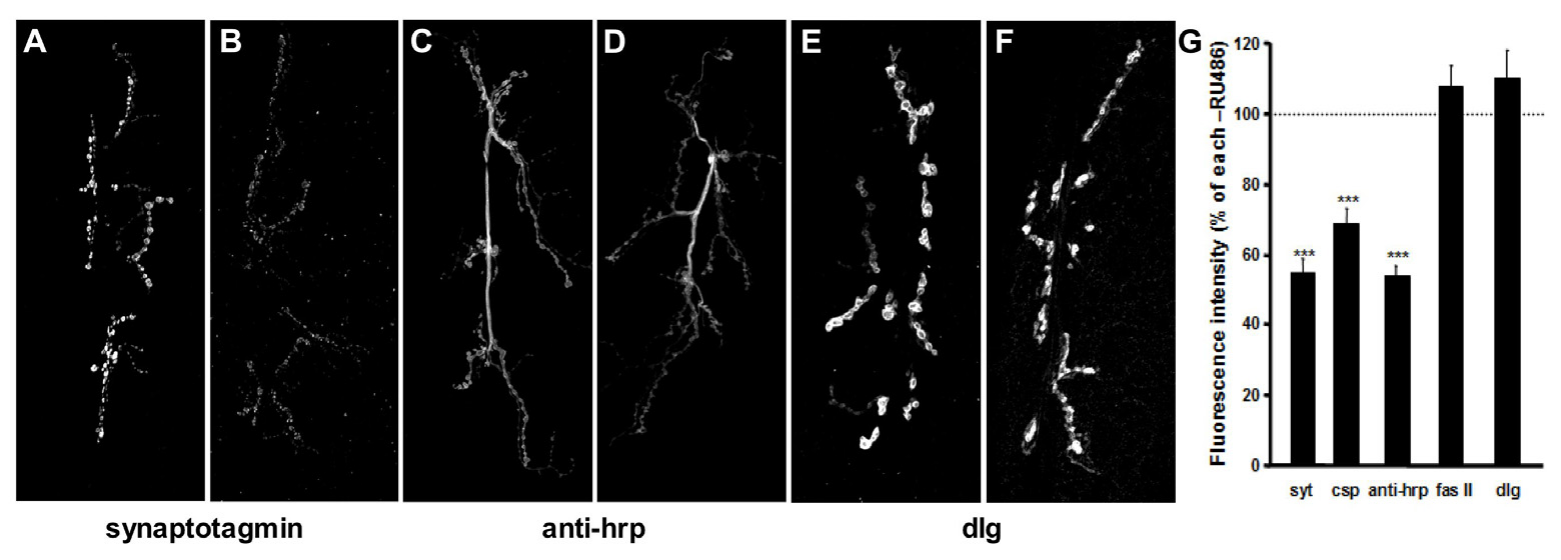
**Presynaptic protein levels decrease with *hep*^act ^overexpression. **Confocal projections of synapses show that levels of presynaptic protein synaptotagmin (Syt) (B) and an antigen recognized by anti-HRP (D) are reduced after *hep*^act ^induction compared to controls (A, C), whereas levels of the postsynaptically enriched protein dlg is similar to control (E) after *hep*^act ^induction (F). G) Quantification of fluorescent intensities show that levels of presynaptic proteins Syt, Csp and anti-HRP, are reduced by 45%, 34% and 50% respectively (*P *< 0.001 for all) compared to control. Levels of postsynaptically enriched proteins FasII and Dlg go not significantly change after *hep*^act ^induction in postembryonic neurons.

Presynaptic proteins including synaptic vesicle proteins, Synaptotagmin, Csp and antigen(s) recognized by anti-HRP were substantially decreased when JNK was activated in motor neurons (Figure 3A–F). Levels of Syt staining were reduced by 45%, Csp by 30% and anti-HRP by 50% (Figure 3G). In contrast, postsynaptically enriched proteins, Fasciclin II and Dlg, do not show any change in intensity.

Because JNK has been implicated in axonal transport, we asked whether transport defects could possibly explain how JNK alters presynaptic composition [43,44]. Defects in axonal cytoskeletal assembly or anterograde axonal transport cause accumulation of Syt positive puncta on axonal tracts [44]. Such organelle jams were not present in axonal tracts of larvae overexpressing *hep*^act^. The nerves were indistinguishable from control animals, indicating that visible axonal transport defects are not present after overexpression of *hep*^act ^(data not shown). For the purposes of this study, these results simply point to potential effects of JNK activation in the CNS that go beyond its previously defined role as a positive regulator of AP-1 and, thereby, of synaptic growth and synaptic strength [35].

### Genome-wide screen to identify JNK targets in neurons

**Figure 4:**
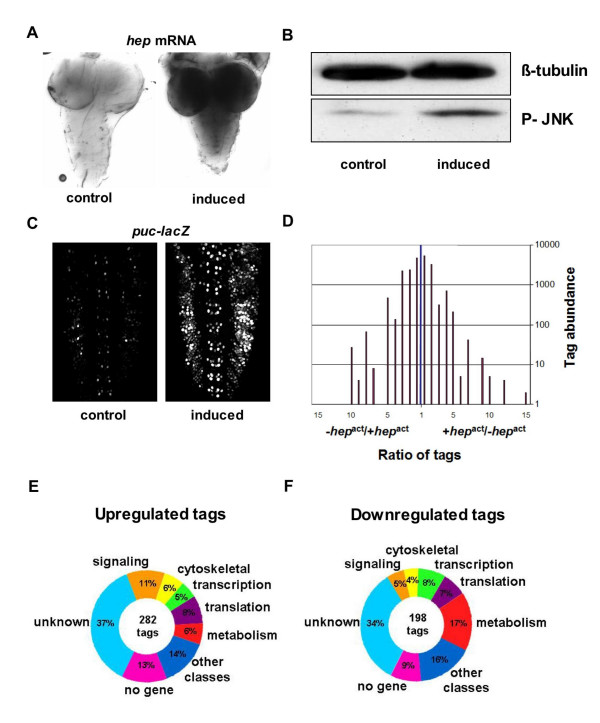
**Acute induction of *hep*^act ^in larval neurons. **A) RNA *in situ *hybridization using a probe specific for *hep *shows inducible expression of *hep *mRNA in third instar larval CNS. B) Western blot analysis of larval CNS protein extracts shows increased levels of activated JNK (P-JNK) after *hep*^act ^induction. C) A lacZ enhancer trap line of *puc *shows increased *lacZ *expression after *hep*^act ^induction in larval CNS. D) A distribution of up- and down- regulated SAGE tags comparing *hep*^act ^induced and control libraries, indicates most tags are present in similar numbers in both the induced and control libraries. E) Functional classification of SAGE tags upregulated after *hep*^act ^induction. Approximately 50% of tags map to genes with no known function and to regions of the genome without an identified gene.

**Figure 5:**
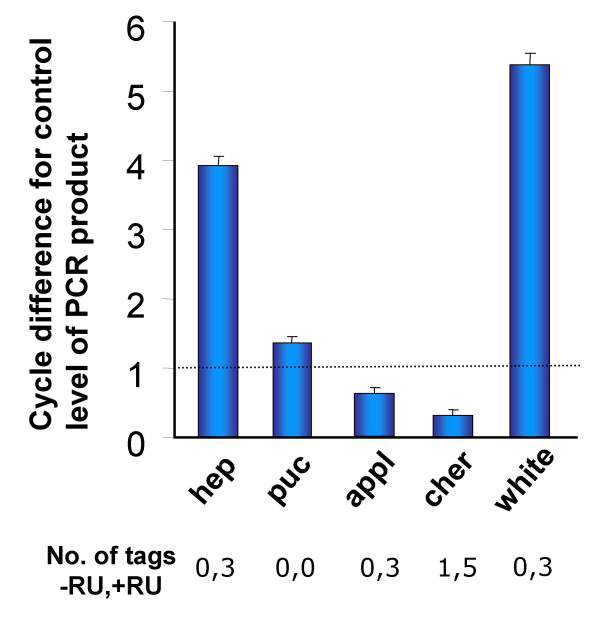
**Q-PCR validation of SAGE results. **Quantitative comparisons of transcript levels in larval CNS RNA from RU486 treated (*hep*^act ^induced) versus control samples. Values represent average cycle difference in PCR product between induced and control samples (N = 5). Each cycle change corresponds to a 2-fold difference in mRNA levels (see Methods). After induction of *hep*^act^, *hep *RNA levels increase 16-fold relative to control (*P *< 0.001); *puc *RNA, not identified by SAGE, is induced three-fold relative to control (*P *< 0.01). While SAGE targets *appl *and *cher *show induction above control RNA levels, *white *is induced more than 32-fold (*P *< 0.001).

To identify transcriptional targets of JNK signaling in the nervous system we performed a genome-wide analysis of JNK-responsive genes in the *Drosophila *larval CNS using Serial Analysis of Gene Expression (SAGE). SAGE is an approach that has been extensively used in analyzing expression changes in cancer cells and other disease states as well as to analyze gene expression in the *Drosophila *embryo and developing eye [45-47]. SAGE is based on generating unique 14 bp tags at a defined position in almost every transcript and, following random sequencing of some 20,000 cDNAs, analyzing the frequency at which each tag (and hence each transcript) occurs in a sample RNA. We used SAGE to a) profile gene expression in the fly central nervous system; and b) identify transcriptional targets of neuronal JNK signaling. To identify early mediators of synaptic change, we analyzed RNA expression 6 hours after JNK activation. We induced a 6-hour burst of neural *hep*^act ^expression in third-instar larval nervous systems using RU486 feeding to induce transcription mediated by neural GS-GAL4. In experimental *ElavGS-GAL4-hep*^act ^animals, we confirmed that JNK signaling was significantly activated by performing the following tests. Quantitative PCR demonstrated a 16-fold induction of *hep *mRNA in larval CNS after *hep*^act ^overexpression (*P *< 0.001) (Figure 5). RNA *in situ *hybridization of RU486 exposed larval CNS showed a marked increase in *hep *mRNA localization in the entire larval CNS (Figure 4A). Immunostaining with an antibody specific for phsophorylated JNK showed induction of *hep*^act ^mRNA leads to activation of JNK (Figure 4B). Finally, we observed that downstream gene expression of a JNK target gene *puc *occurs after *hep*^act ^induction in the CNS. *puc *mRNA is induced nearly 3-fold (*P *< 0.01) by Q-PCR analysis (Figure 5) and in an "enhancer trap" lacZ line we were able to visualize *puc *promoter activity in the larval CNS (Figure 4C). Thus, our protocol to stimulate neural JNK is sufficient to induce an established downstream target of JNK signaling. Exposure of identically cultured, wild-type animals to RU486 did not lead to induction of *hep *or *puc *mRNA or lead to activation of JNK (data not shown). Hence, changes observed between RU486 treated and untreated animals should be largely attributable to JNK signaling in the larval CNS.

**Figure 6:**
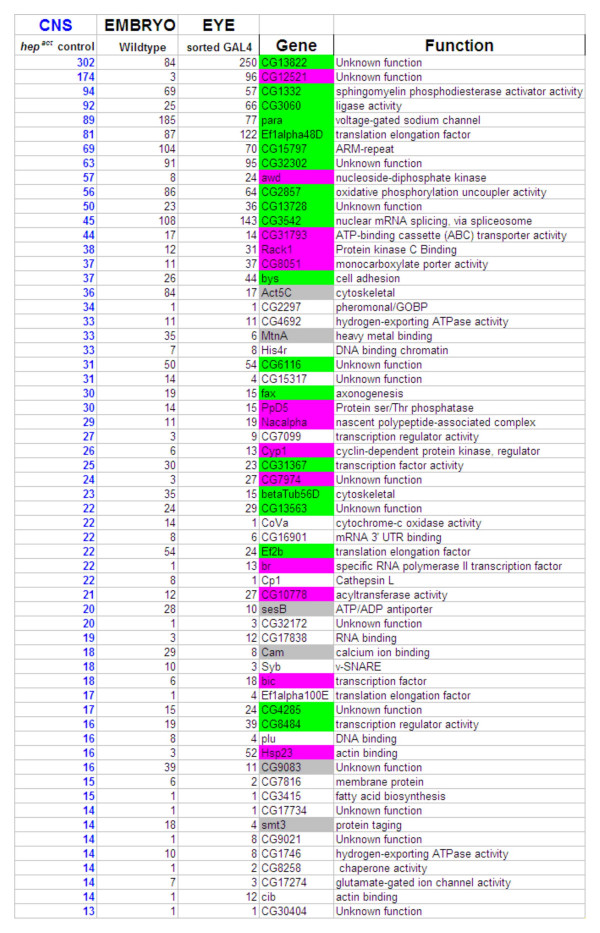
**Comparison of the top 60 expressed genes in larval CNS with expression profiles from embryo and photoreceptor cells identified by SAGE. **The top 60 highly expressed tags from the *hep*^act ^CNS control library were compared with control libraries from embryonic [45] and photoreceptor tissues (Jasper and Bohmann, unpublished data). The number of tags for each gene is indicated on the left for all three tissues and all libraries examined, normalized, to the same number – 20,000 – of total tags sequenced. Tag rankings are sorted in descending order for the control library for each tissue using *hep*^act ^CNS control library as reference, after eliminating tags with more than 3 matches to the genome and excluding any ribosomal RNA binding proteins (highly enriched) and selecting only tags that mapped to an identified gene. 19/60 (green) highly expressed genes in the CNS libraries were also in the top 60 of highly expressed genes in the embryo and photoreceptor libraries while 22/60 (white) are found in the top 60 only in the CNS libraries. There were 13/60 (pink) genes found only in the top 60 of CNS and photoreceptor libraries and 6/60 (gray) genes found only in the top 60 of CNS and embryo libraries.

We sequenced approximately 20,000 tags from individual libraries prepared from dissected larval nervous systems of either control or *hep*^act ^expressing animals. About 9900 unique SAGE tags represented in these libraries were associated with specific genes/genomic sequences using either a database containing predicted tags of all genes annotated by the BDGP [48] or BLAST searches to identify other transcription units [45,47]. Approximately 10% of tags with less than 3 matches to the genome mapped to regions with no predicted gene. About 12.5% of all tags did not match the genome probably due to polymorphisms, errors in sequencing or possible gaps in the published *Drosophila *genome sequence. Comparison of the top 60 expressed genes in the CNS SAGE library to embryonic and photoreceptor SAGE libraries revealed that while 32% of these genes are highly expressed in all 3 tissues such as the cytoskeletal protein *betaTub56D*, 37% are enriched in the nervous system like the translation elongation factor *Ef1alpha100E *(see Figure 6). Such comparisons could prove useful for understanding transcriptional regulation and other processes in different tissues (see Discussion).

Potential JNK-target genes were identified by comparing the relative representation of specific tags in control and *hep*^act ^expressing nervous systems (Figure 4D). A tag was considered up or downregulated when present 3 or more times in a given library and changed at least 3-fold between the two libraries. By these criteria, 346 tags were increased while 271 were decreased following JNK induction. Of these, 25 were "upregulated" and 32 "downregulated" more than 8-fold. Approximately 50% of the induced or repressed tags in the *hep*^act ^library mapped to genes that fell into different functional classes, ~35% of these tags mapped to genes that have no predicted function and ~10% mapped to parts of the genome without any predicted genes (see Figure 4E, F).

To determine whether predictions of SAGE could be confirmed by more careful single gene analyses, we performed Q-PCR to measure relative levels of expression of selected candidate JNK-target genes in control and JNK-induced nervous systems. We selected 61 candidate genes for such Q-PCR verification based on: 1) an abundance of tags for that gene in the *hep*^act ^induced library (9 genes); 2) an interesting known function for the gene (12 genes); 3) presence of AP-1 binding sites in the promoter region of the gene (11 genes); and 4) random selection of genes that did not fall into the above criteria (29 genes). From the 61 genes so examined, 15 that showed induction in at least two independent RT-PCR analyses were analyzed further, namely more extensive Q-PCR analyses using RNAs from 5 independent JNK-induction experiments. In the end, only three genes showed consistent JNK-responsiveness (*P *< 0.05) (Figure 5). *white *showed consistent and robust increases in mRNA levels, whereas *appl *and *cher *showed smaller magnitude inductions. To test if some of the candidate JNK target genes were robustly regulated in subsets of neurons, but diluted out in the Q-PCR analysis, we examined the expression of RNA in the larval CNS using *in situ *hybridizations with probes for several candidate mRNAs. We did not see a clear increase in expression in any of the putative target genes in *hep*^act ^expressing larval CNSs other than *white *(Figure 10; also see microarray screen validation).

The small number of SAGE-predicted JNK target genes confirmed by RNA *in situ *and Q-PCR analyses was difficult to explain without multiple repetitions for which SAGE, being expensive and time-consuming, is not ideally suited. Also, we speculated that genes expressed at lower levels than those identified by SAGE may be true JNK/AP-1 target genes. To test and further these considerations, we used a different genomic approach – oligonucleotide microarrays – to search for neuronal AP-1 target genes.

### Whole-genome microarray screen to identify direct AP-1 target genes in the nervous system

**Figure 7:**
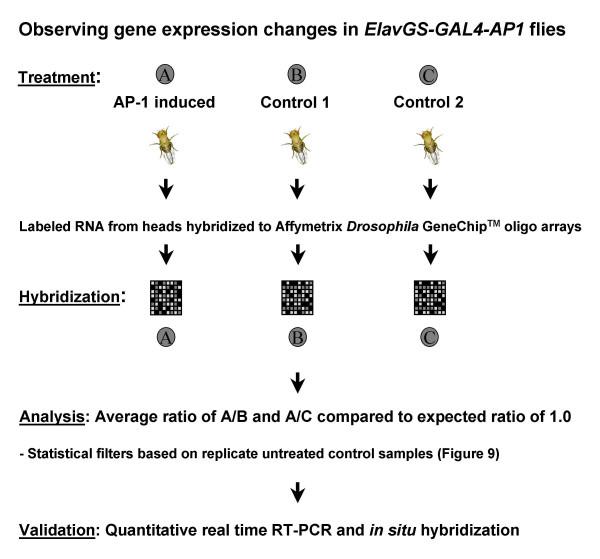
**Microarray experimental design, analysis and validation. **To induce AP-1 in the nervous system, 1–3 day old adult *ElavGS-GAL4-AP1 *flies were treated with the synthetic steroid hormone RU486 in 2% sucrose or sucrose alone for six hrs. Biotinylated RNA from heads was created and hybridized to Affymetrix *Drosophila *Genome1 GeneChip arrays. Gene expression changes between AP-1 induced and control samples were considered significant if they passed a statistical (*P *< 0.01, Student's *t*-test) and secondary filters looking at variance in untreated control samples from the same experiment. Validation of candidate gene expression changes was carried out using quantitative real-time RT-PCR and *in situ *hybridization experiments.

**Figure 8:**
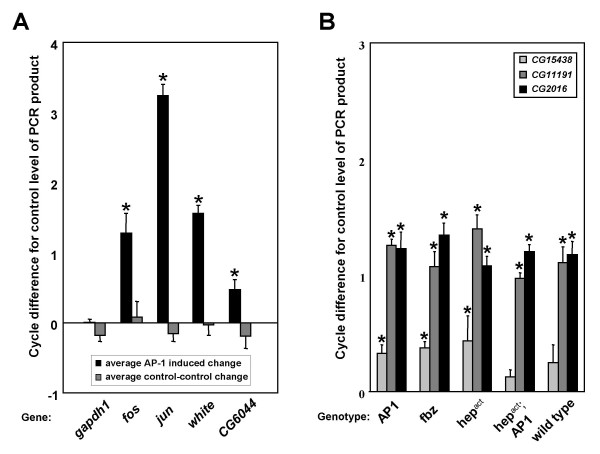
**Q-PCR validation of AP-1 induction and microarray results. **A) Quantitative comparisons of transcript levels in adult head RNA from RU486 treated (AP-1 induced) versus control (black) and control-control (gray) samples. Values represent average cycle difference in PCR product between samples being compared (N = 5). Positive values indicate an increase in transcript compared to unchanging reference gene, negative values a decrease. *fos*, *jun*, *white *and *CG6044 *RNA levels are increased in the fly head after AP-1 induction in the nervous system while untreated control levels show no significant difference. B) Comparisons of transcript levels in adult head RNA from RU486 treated versus control samples. *CG2016*, *CG11191 *and *CG15438 *levels are induced in all samples from flies fed the steroid hormone RU486 (N = 3). X-axis in panel B indicates the UAS-transgene(s) induced by *ElavGS-GAL4 *(AP1: *UAS*-*fos*;*UAS*-*jun*). *Average difference between samples significant at *P *< 0.05 (Student's *t*-test).

We performed comprehensive analyses of transcript levels in fly heads using Affymetrix *Drosophila *Genome1 GeneChip arrays representing the entire annotated genome at the time of its release (~13600 unique genes). An outline of the microarray screen design is illustrated in Figure 7. The analysis compared mRNA levels, with or without AP-1 induction using the same conditional GS-GAL4 strategy described for the previous SAGE analysis. After treatment with RU486 for 6 hours to induce *fos *and *jun *we consistently achieved, respectively, ~2.5- and 9-fold induction of *fos *and *jun *transcripts in fly heads (quantified by Q-PCR in 1–3 day old adult flies, Figure 8A). Untreated control animals showed no significant difference of either gene when levels were compared between age-matched siblings from the same experiment (average untreated change).

Each array "experiment" included sibling flies split into three groups: group A was the experimental (AP-1 induced) population (and groups B and C were independent controls). Thus, each experiment typically allowed transcript levels (normalized hybridization signals) to be compared between experimental and control samples ("A/B" or "A/C" comparisons), and between two identically treated controls (a "B/C" comparison). Candidate AP-1 responsive transcripts would be identified as those with "A/B" and "A/C" ratios significantly different from control "B/C" ratios. This experimental design was useful because hybridization signals for some mRNAs varied significantly more than others and could potentially confound a more straightforward analysis. Through 5–7 repetitions of this basic experiment, we obtained 12 independent experimental versus control ratios, and 5 control-control ratios from which means, variances and SEMs could be determined. Sibling, age-matched controls used in each experiment ensured that genetic background, which can have a large effect on transcriptional variance [49], was not a confounding factor in our analyses.

Based on analyses of 19 hybridizations we established that basic elements of the array technology, probe labeling, hybridization and scanning, were working efficiently and reproducibly (See Methods for a complete description). Microarray hybridization data were passed through three statistical filters to select the most promising AP-1 responsive genes (Methods). Filter 1: We asked that the average ratio of hybridization signal from AP-1 induced versus control mRNA hybridization was significantly (*P *< 0.01) different from 1.0 by Student's *t*-test. Filter 2: We asked that the signal ratio be greater than 1.2. Filter 3: Through analysis of variation observed in identical control-control comparisons, we ensured that genes passing filters 1 and 2 did not show wide variability, for instance based on physiological states of the flies.

**Figure 9:**
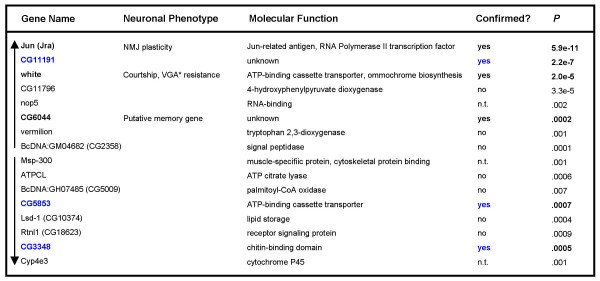
**Top 15 candidate AP-1 responsive genes identified by microarray analysis. **Genes altered following neuronal AP-1 overexpression, passing statistical (Student's *t*-test, *P *< 0.01) and secondary filters based on ratio thresholds and variance in untreated control samples from the same experiment. Arrows on left indicate directionality of expression change listed in order of magnitude from largest positive ratio (induction) on top to largest negative ratio (repression) on bottom. *jun *induction shows the largest expression change by these criteria. Predicted functions from Flybase [70]. Bold type highlights expression changes confirmed by Q-PCR (n.t. = not tested). Blue type highlights RU486-responsive genes. *VGA – volatile general anesthetic.

Using filter 1: of the ~5200 genes considered for analysis (those with relatively strong and specific hybridization signals), 269 showed altered expression after AP-1 induction, with a significance of *P *< 0.01 (Student's *t*-test). Strikingly, 167 genes showed significant upregulation while only 102 were downregulated, a skew consistent with AP-1's expected role as a transcriptional activator. 269 candidates, at *P *< 0.01, is substantially larger than predicted by random chance (52 genes – 0.01 × 5200 genes). However, when a second filter – a requirement that the signal ratio modulus be greater than 1.2 – was applied, the number of candidates dropped to 115. Though small, such signal ratios could correspond to higher mRNA ratios and have been reported as meaningful in previous microarray experiments. Filter 3, to eliminate "variable" genes, trimmed the list of candidate genes that respond consistently to AP-1 overexpression in the fly head to either 4 (*P *< 0.01) or 16 (*P *< 0.05, listed in Figure 9) for which "A/B" and "A/C" ratios were significantly greater than control "B/C" values by Student's *t*-test.

An internal control for the array screen and analysis was provided by *Drosophila *Jun (*Jra*), whose mRNA was experimentally induced. We found that *jun *ranked highest once all three filters were applied and showed robust induction with an average log_2 _treated expression ratio of 1.26 (*P *= 5.9e^-11^) and an average untreated expression change of only -0.07 (Figure 8A). In contrast, *fos *did not pass these stringent filters although we consistently observed an average 2.3-fold increase in *fos *transcript levels by quantitative RT-PCR (Figure 8A). This discrepancy may arise from either of two limitations: a) that *fos *is a low-abundance transcript in the fly head, below the threshold for quantifiable gene expression change detection using Affymetrix GeneChip arrays; or b) the *fos *probe on this particular array may not perform reliably [50], perhaps hybridizing to other non-specific RNA probes. Many gene probes could have similar problems; indeed, other transcripts may exhibit altered expression levels beyond the scope and sensitivity of this assay.

We searched promoters (sequences 3 kb upstream of the translation start sites) of the top 15 candidate AP-1 responsive genes for conserved AP-1 or CREB binding sites and compared their frequencies of occurrence in this group with frequencies observed in a control group of 15 genes that appeared insensitive to AP-1 induction. This analysis revealed no significant enrichment of CREB or AP-1 binding elements in promoters selected based on the microarray experiments (data not shown).

### Microarray screen validation

A major task after initial microarray screening has been completed is confirmation of candidate gene transcript level changes using secondary, independent tests for gene expression. Although the frequency of false-positives is substantially reduced through repetition, a subset of observed expression differences should be validated by other methods.

To confirm positives, a subset of the most robustly changing AP-1-responsive genes, exhibiting significant up- or down-regulation by microarray analysis, were selected as candidates for real-time quantitative RT-PCR validation using gene specific primers (see Figure 9). 12 genes chosen from the group of 15 top candidates mentioned above, in addition to more than 30 genes from outside this stringent set – those with very low "*P*" values or specific predicted biological functions – were selected for these more careful confirmatory experiments. Increases in transcript levels following AP-1 overexpression, detected by Q-PCR, for *fos, jun *and 2 confirmed candidate genes (*white *and *CG6044*) are shown in Figure 8A. All mRNA levels are normalized to the control gene *rp49*. Transcript levels for a second control gene, *gapdh1*, are shown to demonstrate its levels do not change significantly by either AP-1 induced versus control ("A/B", "A/C") or control-control ("B/C") comparisons.

Five uncharacterized genes (*CG2016*, *CG11191*, *CG15438, CG5853 *and *CG3348*) were confirmed by Q-PCR to be consistently altered in RU486-treated, AP-1 induced samples (Figure 9, Figure 8B – data for *CG5853 *and *CG3348 *not shown). In addition, overexpression of *fbz *with RU486 treatment also caused a similar change in transcript levels of these 5 genes. When treated with the steroid, wild type flies and all other transgenic lines tested showed consistent alterations of these 4 mRNA transcripts in the head, suggesting they are hormone-responsive genes in the fly.

**Figure 10:**
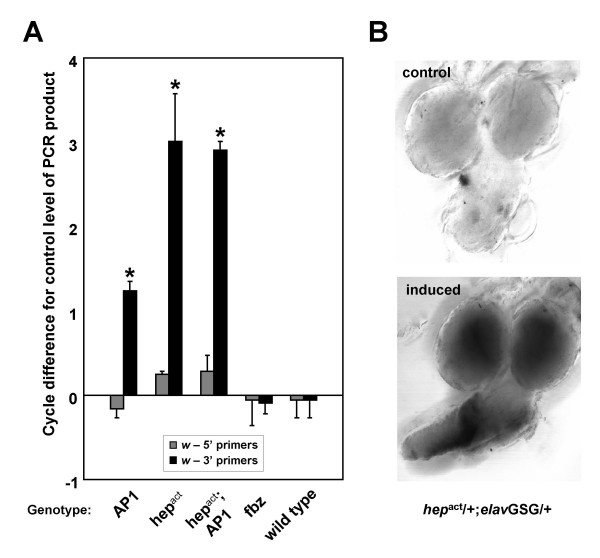
***white *transcript levels are induced when positive JNK pathway components are overexpressed in the fly nervous system. **A) Quantitative comparisons of *white *transcript levels in adult head RNA from RU486 treated versus control samples. PCR primers designed to the 3' end of *white *(black), but not 5' primers (gray), show increased levels in response to AP-1 and *hep*^act ^induction (N = 3). Wild-type flies exposed to hormone or flies overexpressing *fbz *do not show induction of *white*. X-axis indicates the UAS-transgene(s) induced. *Average treated difference significant at *P *< 0.05 (Student's *t*-test). B)*in situ *confirmation of increase in *white *transcript levels following *hep*^act ^induction in the larval CNS.

As in the larval CNS, Q-PCR experiments confirmed *white *gene induction in the adult fly head. *white *transcript levels are significantly increased in the head following AP-1 overexpression in the brain (Figure 8A, Figure 9, Figure 10). Further Q-PCR experiments demonstrated *white *transcripts are increased to an even greater extent when *hep*^act ^is induced in combination with AP-1 or by itself in the adult nervous system (Figure 10A), although its levels are not increased to the degree seen in the larval CNS (Figure 5). *white *is not induced when *fbz *is overexpressed, nor in wild type flies treated with RU486. Only primers designed to the 3' portion of the *white *transcript showed altered levels (Figure 10A), which is consistent with the background strain used in all the experiments(*w*^1118^). This strain lacks the 5' portion of the *white *gene locus [51], yet still contains sequence for and expresses the second through fifth exons (data not shown) that are induced in response to JNK signaling in the fly head. The same transcriptional induction profile is observed in *ElavGS-GAL4-hep*^act ^flies with the wild-type (*w*^+^) copy of the *white *gene on the X chromosome as well as in a *white *null (*w*^11E4 ^[52]) background (data not shown). This suggests *white *induction occurs via the *mini-white *cassette present in pUAST transgenes. RNA *in situ *hybridization experiments confirmed the increase in *mini-white *transcript levels in the CNS of larvae in which *hep*^act ^has been induced (Figure 10B). Increases were also observed in larvae overexpressing AP-1 (data not shown), albeit with smaller magnitude changes consistent with our findings from Q-PCR analyses in the adult head (see Figure 10A).

*CG6044 *induction following AP-1 overexpression was also confirmed in independent Q-PCR experiments (Figure 8A). Consistent increases in transcript levels were observed in all AP-1 overexpressing heads but not in treated *fbz *or wild type heads (data not shown). The induction observed by quantitative RT-PCR and microarray experiments was not reflected in follow-up *in situ *experiments; however, this is likely because the small magnitude increases in transcript levels observed by other means (1.2-fold – microarray; 1.4-fold – Q-PCR) are below the detection range for this method.

## Discussion

This extensive study makes three contributions: (A) it demonstrates unexpected and novel interactions between JNK and cellular processes that underlie synapse plasticity; (B) by SAGE analyses, it provides a genomic profile of mRNAs expressed in the fly larval nervous system; (C) it presents two large-scale genomic approaches to identify JNK and AP-1 targets in the fly CNS providing useful data pertinent to JNK/AP-1 signaling in neurons as well as to genomic analyses in the *Drosophila *nervous system.

### Effects of JNK activation in postembryonic motorneruons

The immediate-early transcription factor AP-1 positively regulates both synapse size and synapse strength at the *Drosophila *larval NMJ [35]. While JNK signaling is necessary for the effect of AP-1 on synapse structure and function, it is not clear whether JNK signaling is sufficient for synaptic change. We show, first, that activation of JNK in post-embryonic neurons leads to significant synaptic alterations; second, that these alterations are inconsistent with JNK functioning solely through AP-1. Our finding that activation of JNK signaling leads to an increase in synapse number but decreases synapse strength indicates that JNK activates not only AP-1, a positive regulator of growth and strength, but also a pathway that negatively influences synaptic strength.

### The neural transcriptome, and its regulation by JNK and AP-1

The ability of SAGE to evaluate absolute expression levels of gene transcripts enables relatively facile, quantitative, profiling of gene expression in any given tissue (or RNA source). Given the intense interest in *Drosophila *neurobiology, a previous painstaking sequence analysis of some 1000 cDNAs from a fly brain cDNA library provided useful new information on the neural transcriptome [53]. The analysis presented here, following sequencing of about 20,000 ESTs from two independent brain libraries, substantially extends the previous study. The use of this resource is demonstrated by our simple survey of highly expressed neuronal RNA-binding proteins, potentially involved in important neural-specific, post-transcriptional functions such as translational repression, mRNA transport or RNA editing. 10% of the 60 most highly expressed (non-ribosomal) mRNAs in nervous system encode RNA-binding proteins, 2 of which are enriched in neurons versus embryonic tissue. A significant fraction of these (3/6) have conserved homologs recently found on RNA granules, organelles containing translationally repressed mRNAs which are actively transported to synaptic sites [54]. We have recently begun functional analyses of some of these RNA-binding proteins. Similarly, we anticipate that identification of tissue-specific genes could provide unanticipated launch points for investigation into their cellular functions.

Given the evidence to indicate wide effects of AP-1 and JNK on synaptic properties, we searched for AP-1 and JNK-target genes using both SAGE and microarray approaches to determine effects of JNK and AP-1 signaling on neuronal gene expression. Of the two approaches, microarray analysis, being dependent on parameters such as hybridization and labeling efficiencies that vary among individual transcripts, is not ideal for quantitative analyses as outlined in the previous section. However, it provides information on transcripts with low to moderate levels of expression, is fast, and allows multiple iterations of each experiment at a small cost relative to SAGE.

In order to identify early transcriptional targets, most likely to link JNK and AP-1 activation to synaptic change, we used the steroid-inducible GAL4 system, an increasingly popular strategy to achieve conditional, tissue-specific transgene expression in *Drosophila *[35,41,55,56]. SAGE-derived transcript profiles of RNA extracted from whole larval CNSs showed several potentially significant targets. However, very few were confirmed by secondary low-throughput, gene-specific analyses. Microarray-derived transcript profiles of adult head mRNA showed similar results. Several statistically significant targets of AP-1 signaling were initially identified; however, few were confirmed by carefully controlled application of the most commonly used transcript-specific analyses (quantitative RT-PCR and *in situ *RNA hybridizations). While the implications of these results for neurogenomics are briefly discussed in the next section, we first consider the "positive" genes identified by SAGE and microarray screens.

Quantitative RT-PCR validation of the generated SAGE data resulted in the identification of 3 genes, *cher*, *appl *and *white*, which were consistently upregulated following JNK activation in the larval CNS. Though we were unable to evaluate induction of *cher *and *appl *by RNA *in situ *hybridization, *white *showed robust increases by this method as it did by Q-PCR. A total of seven expression changes identified in the microarray screen were verified by Q-PCR analysis; remarkably, five turned out to be genes responding to RU486 treatment itself rather than to consequent AP-1 induction. These steroid-responsive genes may be of significant biological interest. However, from our point of view they serve primarily to: a) further establish the bonafides of our experimental and analytical protocols; and b) as a useful caution for Drosophilists and others using the steroid-inducible conditional expression system. The remaining two confirmed AP-1 target genes were w *hite*, also identified in the SAGE screen but shown eventually to be expressed from the P-element associated *mini-white *locus, and *CG6044*. Of potential significance, *CG6044 *has been implicated in olfactory associative memory [40].

AP-1 responsiveness of *CG6044 *was verified in Q-PCR validation experiments (Figure 8). The gene was previously found in a mutational screen for putative memory genes required for normal olfactory conditioning in *Drosophila *[40]. In addition, it is one of the few genes from the list of likely AP-1 targets (listed in Figure 9) that has a conserved AP-1 binding site within 500 base pairs of its translation start site. It is therefore a promising candidate warranting further investigation into the role it plays in synaptic plasticity and memory formation.

### Lessons and limitations

It appears unlikely, if not inconceivable, that the 4 probable downstream genes enumerated above could mediate the demonstrated effects of AP-1 or JNK induction on motor-synapse properties. Thus, the genomic approaches we have followed, while informative, have likely not led to the identification of JNK/AP-1 targets that link these signals to synaptic change. One possible interpretation, that the experiments were technically flawed, appears to be ruled out, not only because internal controls (Jun, Hep, Puckered and steroid-responsive genes) were identified in the screens, but also because various standards for microarray hybridization data and SAGE library complexity were evaluated and shown to be well within the technically optimal range. Thus, we are left with the second interpretation, that analysis of whole-brain mRNA may not allow targets of signaling pathways to be unambiguously identified. A major issue is likely to be cell-type heterogeneity within the brain. If different subsets of neurons show substantially different genomic responses to JNK/AP-1 (including the absence of a response), then altered expression of the meaningful JNK/AP-1 targets in a subset of cells may be diluted by the large background of mRNA deriving from other neuronal types.

At a conceptual level, Barolo and Posakony have nicely articulated the concept of "activator insufficiency" and the need for cooperative activation of multiple transcription factors for turning on transcriptional pathways governing developmental processes [57]. Considerable evidence argues that neurons are a diverse class of cells with a range of distinct transcriptional ground states. For example, cell-type-specific binding of CREB to known target gene promoters has been shown in various cell types under basal and stimulated conditions [58]. Similarly, the response of different neuronal populations to TGFβ has been shown to be highly context dependent and to derive from variations in expression of specific TGFβ insensitive transcription factors [59]. Thus, genomic analyses when applied to whole nervous systems may have significant intrinsic limitations.

Nevertheless, some conserved downstream genes may still be revealed [60-63]. For instance, the steroid hormone, RU486, used to induce transgene expression in our experiments presumably activates a set of hormone-responsive genes in a large subset of neural cells. However, for incisive mechanistic analyses for which *Drosophila *is so convenient, we suggest that genome-wide screens described to study signaling responses in the nervous system be applied with specific refinements, such as emerging methodologies to prepare sufficient mRNA from a homogeneous population of cells in which biological function of these signaling pathways have been evaluated [64]. Various GFP transgene lines should make it possible to sort specific cell populations prior to genomic screens to identify transcriptional targets.

The availability of new genetic and molecular tools and refined functional genomic approaches should result in continued understanding of how kinases and transcription factors regulate molecular changes that occur in the *Drosophila *nervous system, as well as intrinsic flexibility and constraints of these signaling pathways.

## Conclusion

This study revealed unexpected relationships between JNK signaling and synaptic plasticity in *Drosophila *that are inconsistent with a role for JNK acting solely through AP-1 to affect strength of the synapse. It also presents a profile of the transcriptome of the larval nervous system and, while providing potential transcriptional targets of JNK and AP-1 signaling in neurons, points out the pitfalls of genome-wide analyses in complex tissues such as the whole fly nervous system.

## Methods

### Fly strains and genetics

We used the following strains: wild type (Oregon R; D. Brower); GAL4-responsive *UAS-hep*^act ^(M. Mlodzik), *UAS-fbz, UAS-fos, UAS-jun *(M. Bienz), *puc-lacZ *line – *puc*^*e*69^(A. Martinez Arias); neural GAL4 lines – *C155, C380, D42 and OK6 *were from C. Goodman, V. Budnik, G. Boulianne and B McCabe, respectively; *ElavGS-GAL4 *line was from T. Osterwalder and H. Keshishian.

### Postembryonic and acute induction in neurons

#### Induction in larvae

All animals were generated by crossing males homozygous for UAS-transgenes (or wild-type males) with virgin females homozygous for the *ElavGS-Gal4 *driver. All animals were raised at 25°C and parents transferred to a new vial each day for age-selection of larval instar stages. For postembryonic induction, larvae in vials that should contain a majority of late 1^st ^instar-early 2^nd ^instar were transferred into a standard vial containing 0.015 mg/ml RU486 (Sigma) for 48 hrs before climbing third instar larvae were selected for further analysis. Control animals were exposed to food containing only 4% ethanol (same as treated) and analyzed accordingly. For acute induction in 3^rd ^instar larvae, age-selected larvae were transferred to a 1.5 ml sample tube containing 0.5 ml of 3 mg/ml RU486 for 2 min, before they were washed and transferred into a standard vial containing 0.015 mg/ml RU486 (Sigma) for 6 hrs before the CNS was dissected for further analysis.

#### Induction in adults

As in larvae, all animals were generated by crossing males homozygous for UAS-transgene constructs (or wild-type males) with virgin females homozygous for the *ElavGS-GAL4 *driver. Progeny reared at 25°C were aged to be 1–3 days old at time of treatment. 16–64 hour old adults were starved for 8 hours in a Tupperware container filled with desiccant to keep the humidity level at ~16% and ensure ingestion of the treatment medium. Flies were then split into separate bottles and fed for 6 hours. Each treatment consisted of *ElavGS-GAL4-UAS *flies handled identically (aged and starved in the same bottle) except RU486 was added to the sucrose fed to the experimental group. Experimental animals were fed on a kimwipe soaked with RU486 in 2% sucrose at a final concentration of 0.04 mg/ml, taped to the bottom of a large, dry, empty bottle. Sibling control flies of the same genotype were fed sucrose alone.

### Immunostaining

Larvae were raised at 25°C after postembryonic induction of *UAS *transgenes, dissected, stained with anti-Syt antibody and mounted. Bouton number was counted from projections of confocal sections at 60X magnification. Boutons at segment A2 in muscle 6 and 7 were manually counted without knowledge of the genotype (blind counting), using Metamorph imaging software. No significant difference in muscle surface area, measured using a drawing tool in Metamorph was observed in the different genotypes. To quantify levels of synaptic proteins synapses labeled with specific antibodies; anti-syt, anti-csp, anti-HRP, anti-fas II, anti-dlg, were identically imaged for control and induced animals and the average pixel intensity of terminal boutons (3–4) was measured and analyzed. To quantify organelle accumulation on axons after *hep*^act ^induction, larval segmental nerves were imaged at high resolution using a cooled charge-coupled device camera (Princeton Instruments) and Metamorph imaging software (Universal Imaging). After background subtraction, images were analyzed for organelle jams and compared with control animals.

### Electrophysiology

All electrophysiological recordings were made from muscle 6 within A2, with the larval preparation immersed in a low volume of the HL3 saline with 1 mM Ca^2+^. Electrophysiology was performed as described previously. In all experiments, the CNS was gently removed to prevent endogenous motor firing. Motor nerves were stimulated with glass-tipped suction electrodes. For intracellular recordings, electrodes pulled from borosilicate capillary tubes were backfilled with 3 M KCl, yielding resistances of 6–10 MΩ. To ensure good recordings, preparations with resting potentials more positive than -60 mV were discarded. For recording excitatory junctional potentials (EJPs), an isolated pulse stimulator (A-M systems, Everett, WA) was used to deliver 1 msec pulses at a frequency of 1 Hz to elicit an evoked response. All recordings were acquired with an axoclamp 2B amplifier in conjunction with pClamp 6 software (Axon Instruments, Foster City, CA). The EJP amplitude for each preparation was determined from an average of 15 consecutive evoked responses. For quantifying mini frequencies, the number of mEJPs occurring consecutively within 30 sec was counted for each preparation. The mEJP amplitude for each preparation was determined from an average of 30 consecutive mEJPs. At least 5 animals were analyzed for each genotype. For each animal examined that was exposed to RU486 treated food, we examined control animals and expressed the quantal content of transmitter release as a percentage of control.

### Serial Analysis of Gene Expression (SAGE)

SAGE was performed as previously described [45,47]. Briefly, polyA mRNA from 50 CNSs dissected out of drug treated 3^rd ^instar larvae was purified with dynabeads mRNA direct kit (Dynal). Double-stranded cDNA was synthesized on the beads and digested with the anchoring enzyme (NlaIII; NEB). After linker ligation, digestion with the tagging enzyme (BsmFI, NEB), and ligation of the ditags, PCR amplification (29 cycles) was carried out with 20% of the ligation product as template. The 100 bp PCR products were purified and submitted to a secondary PCR (10–12 cycles) with biotinylated primers to generate enough material for the concatemerization. After NlaIII digestion, the released ditags were purified by polyacrylamide gel electrophoresis and subsequently incubated with 100 *u*l of Dynabeads Streptavidin to eliminate any remaining biotinylated linkers. Concatemerization was carried out for four hours. Concatemers were cloned into the SphI site of pZero1 (Invitrogen), and resulting colonies were screened for inserts by PCR and submitted for sequencing. All sequencing reactions and SAGE tag generation was performed at Agencourt Inc. (Boston).

### Analysis of SAGE Data and Annotation of SAGE Tags

Sequenced SAGE concatemers were analyzed using the SAGE2000 program obtained from The Johns Hopkins University (see also [65]). The database linking SAGE tags to data of the Berkeley *Drosophila *Genome Project was built using datasets downloaded from the BDGP site [66] and extracting the 10 bp sequence downstream of the 3'-most CATG site. These putative tags were linked to the GadFly site of the corresponding gene. Annotation of experimental data was performed using Microsoft Access to link the experimental dataset and the Tag annotation database [45,47].

### Microarray analysis

Total RNA was extracted from 200–300 heads of RU486-treated (AP-1 induced) and untreated control *ElavGS-GAL4-AP1 *flies (*w*^1118^;*UAS-fos*/+;*UAS-jun*/*ElavGS-GAL4*) using the RNeasy kit (Qiagen). 5 *u*g of total RNA was used as a starting template for 19 microarray hybridizations (7 treated and 12 untreated RNA samples). Two sets of control flies were included for analysis in five of the seven experimental AP-1 overexpression treatments used for the microarray hybridizations. Transcript quantification was performed with Affymetrix *Drosophila *Genome1 GeneChip [67] arrays using biotinylated cRNA targets prepared according to standard Affymetrix protocols by the GATC Affymetrix Core Facility at the University of Arizona [68]. Hybridized arrays were scanned using Affymetrix MicroArraySuite software as described in the manufacturer's protocol. All hybridizations were normalized with a global scaling factor of 500 so that transcript levels could be compared directly. Text files containing raw, normalized values were exported into Excel for further analysis.

Internal control, 3'-5' probe signal ratios (a measure of how well the biochemical reactions went prior to hybridization of the biotinylated probe to the oligonucleotide array) were within the range recommended by the manufacturer for all hybridizations. R^2 ^values for all comparisons of control versus control samples from the same experimental group were high (≥ 0.97).

Between 41% and 49% of all genes were scored present or marginal on each of the arrays by MicroArraySuite. In order to avoid spurious data, only the 5188 genes present or marginal in all 7 AP-1 induced samples were considered for further analysis (~38% of all probes on the array). Ratios between AP-1 induced and the 1 or 2 control samples from a given treatment were calculated in Excel. Fold-differences were converted to log_2 _values so that increasing and decreasing levels of mRNA could be compared directly. Log_2 _values (n = 12) were tested against the value of 0, expected if there were no change in expression, using the Student's *t*-test (unpaired *t*-test, two-sided *P*, samples with unequal variance estimates). The *P-*values accepted for our analysis (*P *< 0.01) therefore reflect a 99% probability that the null hypothesis (there is no difference in the expression of a given transcript in AP-1 induced samples) should be rejected.

Secondary filters to eliminate false positives and randomly fluctuating transcripts included: 1) the average AP-1 induced versus control ratio (n = 12) for a given gene had to be 1.2 or higher; 2) Log_2 _values for the expression ratio, comparing AP-1 induced to control signals for a given gene (n = 12), were tested against the values of control versus control ratios (n = 5), again using the Student's *t*-test – genes passing this statistical filter (*P *< 0.05) were considered to be changed beyond the dynamic nature of the transcript.

### Quantitative real time RT-PCR and *in situ *hybridization

#### Larval CNS

To quantify RNA expression, approximately, 25 larval brains were dissected for each sample. PolyA mRNA was isolated using the Dynabeads mRNA direct kit (Dynal) and oligo dT-primed cDNA was synthesized with the Omniscript cDNA synthesis kit (Qiagen). The cDNA was diluted 1:5 for Q-PCR reactions performed on a Cepheid SMARTCycler using QuantiTect SYBR Green PCR kit (Qiagen). Transcript levels were determined using gene-specific primer sets (details available on request). Expression differences are shown as the average change in cycle number at which PCR product (determined by fluorescent signal) is detected as statistically significant above background. This is referred to as the crossing threshold and the more cDNA template present at the start of the reaction, the fewer number of cycles it takes to reach this point. A one-cycle difference represents a two-fold difference in starting template concentration. All transcript levels are normalized to the control gene, ribosomal protein 49 (*rp49*), as previously described [35].

#### Adult heads

Independent RNA samples were extracted as for microarray experiments for all Q-PCR comparisons. Equal amounts of total RNA (4 *u*g) for RU486-treated (induced) and untreated control samples were purified from genomic DNA with the DNA-free DNase kit (Ambion) prior to oligodT-primed cDNA synthesis using the Omniscript cDNA synthesis kit (Qiagen). The cDNA was diluted 1:20 with nuclease-free H_2_O (Invitrogen) for Q-PCR reactions performed as described above. Each PCR reaction was repeated in triplicate for 3–5 independent RNA preparations from separate RU486 treatments. Sample sets were compared using the Student's *t*-test as for the array analysis and only results showing a *P*-value <0.05 were considered statistically significant.

*In situ *hybridizations were performed using probes prepared with PCR DNA (400–600 bp) from primers specific for gene of interest containing T7 RNA polymerase binding site in the sense orientation and SP6 RNA polymerase-binding site in the antisense orientation. RNA probes were labeled with DIG and visualized using either NBT/BCIP (blue reaction product). Larval CNSs were dissected after drug treatment and the tissue was processed using standard protocols [69].

## Authors' contributions

PDE and MR conceived of and designed the AP-1 overexpression experiments. PDE performed the microarray experiments and statistical analysis; PDE and CP performed the microarray confirmation experiments. RN, HJ, MR and DB conceived of and designed the SAGE experiments. RN and HJ performed the SAGE experiments and analysis; RN and ZN performed the *hep*^act ^overexpression and SAGE validation experiments. PDE, RN and MR drafted the manuscript with input from the other authors.
